# Biotransformation of Endocrine-Disrupting Compounds in Groundwater: Bisphenol A, Nonylphenol, Ethynylestradiol and Triclosan by a Laccase Cocktail from *Pycnoporus sanguineus* CS43

**DOI:** 10.1007/s11270-015-2514-3

**Published:** 2015-07-10

**Authors:** R. Garcia-Morales, M. Rodríguez-Delgado, K. Gomez-Mariscal, C. Orona-Navar, C. Hernandez-Luna, E. Torres, R. Parra, D. Cárdenas-Chávez, J. Mahlknecht, N. Ornelas-Soto

**Affiliations:** Centro del Agua para América Latina y el Caribe, Tecnológico de Monterrey, Monterrey, NL 64849 Mexico; Universidad Juárez Autónoma de Tabasco, Av. Universidad S/N Magisterial, Villahermosa, 86040 Tabasco Mexico; Laboratorio de Enzimología, Facultad de Ciencias Biológicas, Universidad Autónoma de Nuevo León. Av. Universidad s/n, Ciudad Universitaria San Nicolás de los Garza, San Nicolás de los Garza, NL 64450 Mexico; Posgrado en Ciencias Ambientales, Benemérita Universidad Autónoma de Puebla, Edificio 103G, 7, Puebla, Mexico

**Keywords:** Laccases, *Pycnoporus sanguineus*, Endocrine disrupting compound (EDC), Groundwater

## Abstract

The biodegradation of organic compounds present in water at trace concentration has become a critical environmental problem. In particular, enzymatic oxidation by fungal laccases offers a promising alternative for efficient and sustainable removal of organic pollutants in water. In this work, the biocatalytic ability of laccases from the *Pycnoporus sanguineus* CS43 fungus was evaluated. A filtered culture supernatant (laccase cocktail) evidenced an enhanced biotransformation capability to remove common endocrine-disruptor compounds (EDCs), such as bisphenol A, 4-nonylphenol, 17-α-ethynylestradiol and triclosan. A biodegradation of around 89–100 % was achieved for all EDCs using synthetic samples (10 mg L^−1^) and after the enzymatic treatment with 100 U L^−1^ (50.3 U mg ^−1^). The biodegradation rates obtained were fitted to a first order reaction. Furthermore, enzymatic biocatalytic activity was also evaluated in groundwater samples coming from northwestern Mexico, reaching biotransformation percentages between 55 and 93 % for all tested compounds. As far as we know this is the first study on real groundwater samples in which the enzymatic degradation of target EDCs by a laccase cocktail from any strain of *Pycnoporus sanguineus* was evaluated. In comparison with purified laccases, the use of cocktail offers operational advantages since additional purification steps can be avoided.

## Introduction

Over the last few decades, the water pollution by micropollutants from anthropogenic sources, such as pharmaceuticals, manufacturing additives or personal-care products, has become one of the most urgent issues to be solved (Lloret et al. 2012). Due to their widespread presence in the environment and their toxic activity even at low concentrations (pgL^−1^ – ngL^−1^; Debaste et al. 2014), the detection and quantification of endocrine-disruptor compounds (EDCs) have received increased attention in water quality management and health care, since their presence has been detected in rivers, lakes, groundwater (Sacher et al. 2001; Vega et al. 2007) and other sources of drinkable water (Robert et al. 2011; Stanford and Weinberg 2007).

**Fig. 1:**
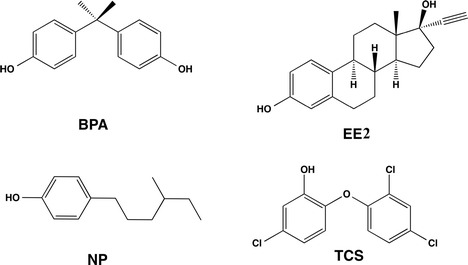
Schematic structures of target EDCs: bisphenol A (BPA), 17-α-ethynylynyl estradiol (EE2), 4-nonylphenol (NP) and triclosan (TCS)

EDCs are a group of environmental chemicals, from synthetic and natural origin, known for their negative influence on the endocrine system of living organisms (LaFleur and Schug 2011). Several studies have demonstrated that these chemicals mimic hormones or interfere with the action of endogenous hormones (Filby et al. 2007; Cabana et al. 2007a). Removal of EDCs traces in wastewater treatment plants (WWTP) has been a constant challenge due to higher concentrations that can remain in the treated waters. The accumulation of EDCs in the environment has been linked to cancer proliferation, mutations and reproduction disruption in fish, amphibians, birds and mammals, including humans (LaFleur and Schug 2011). Typical EDCs of anthropogenic origin with estrogen-like action include bisphenol A (BPA), 4-nonylphenol (NP) and 17-α-ethynylynyl estradiol (EE2); (Cajthaml et al. 2009), as well as antibacterial triclosan (TCS); (see Fig. 1). Due to EDCs that are slowly biodegraded under aerobic conditions, some of them can persist for more than 40 years as observed in estuary sediments (Miller et al. 2008).

Under such circumstances, the increasing accumulation of EDCs and bactericides in the environment has motivated the investigation of methods with the ability to reduce or inactivate these chemicals. Previous works have been directed on UV exposure as well as advanced oxidation processes such as ozonation (Esplugas et al. 2007). Although these methods produce efficient yields of removal/inactivation, they are expensive and may generate by-products with higher toxicity (Lloret et al. 2012). A promising approach to overcome these limitations is oxidation of EDCs by employing ligninolytic enzymes such as manganese peroxidase and laccases (Suzuki et al. 2003). The use of fungal laccases (blue copper polyphenoloxidase, E.C.1.10.3.2) offers a high potential to degrade and detoxify recalcitrant environmental pollutants (Tsutsumi et al. 2001; Torres et al. 2003).

An exhaustive search concerning laccases from *Pycnoporus sanguineus* was carried out, and to the best of our knowledge, there are no previous studies about the transformation of BPA, NP, EE2 and TCS using this strain in particular. Moreover, it was found that only a few works (Tsutsumi et al. 2001; Saito et al. 2004; Cabana et al. 2007a; Torres-Duarte et al. 2012) have studied the biotransformation of the target EDCs simultaneously with laccases from other commercial strains.

Due to the biotransformation, efficiency of organic compounds by ligninolytic enzymes in water represents an interesting option for environmental and industrial applications; in this work, a filtered culture supernatant containing a cocktail of laccases from *P. sanguineus* CS43 was assayed with synthetic and groundwater samples coming from northwestern Mexico. In order to establish a sustainable methodology for the biodegradation of BPA, NP, EE2 and TCS, a treatment avoiding the use of mediators and under mild conditions was developed.

## Experimental Section

### Enzyme Laccase and Reagents

Laccases from *P. sanguineus* CS43 were obtained from a tomato medium as described in our previous study (Ramírez-Cavazos et al. 2014a). Mycelia were removed from the culture supernatant by filtration using two tangential flow filters in series, with pore sizes 0.5 and 0.2 μm. After that, the 0.2-μm filtered culture supernatant (laccase cocktail) was ultra-filtered by using a membrane cut-off of 10 kDa. The ultrafiltration process avoids the presence of lower molecular weight solutes present in the culture that can represent an environmental risk. Standards of BPA, NP, EE2 and TCS (high purity grade), 2,2′-azino-bis (3-ethylbenzthiazoline-6-sulfonate (ABTS), dibasic sodium phosphate and citric acid salt were obtained from Sigma Aldrich, USA. Methanol, acetonitrile and ethanol (trace analysis quality) were supplied from Fisher Scientific, Tedia and Fermont, respectively.

### Enzyme Characterization

The presence of two abundant laccase isoforms, denominated Lac I and Lac II, in the filtered culture supernatant (laccase cocktail) obtained from a tomato medium is described in our previous work (Ramírez-Cavazos et al. 2014b) To establish the conditions of biodegradation on the target EDCs (i.e. BPA, NP, EE2 and TCS), the purification, characterization and stability information of the two laccase isoforms were applied and used as previously described.

### Enzymatic Activity Assay

Spectrophotometric measurements were performed in a micro-plate reader Omega FLUOstar. The enzyme activity of 20 μL aliquots of appropriately diluted laccase cocktail or purified enzyme was assayed with 5 mM ABTS as the substrate in pH 3 buffer McIlvain (0.2 M sodium phosphate dibasic/citric acid 0.1 M), 25 °C, at 420 nm (ɛ_nm_ = 36,000 M^−1^ cm^−1^). The enzyme activities were expressed as international units (U), defined as the amount of enzyme necessary to produce 1 μmol of product formed per minute.

### Enzymatic Treatment

Stock solutions of 100 mg L^−1^ were prepared by dissolution of the standards in a mixture of ultrapure water–ethanol 50–50 % (*v*/*v*). Enzymatic reactions were carried out in 10 % (*v*/*v*) McIlvaine buffer (dibasic sodium phosphate 0.2 M/citric acid 0.1 M) pH 5 containing 10 mg L^−1^ of each analyte (using aliquots from stock solutions). Reactions were performed at room temperature and started by adding 100 U L^−1^ (50.3 U mg ^−1^) of laccase. In order to perform a qualitative and quantitative biodegradation of EDCs, UV–vis spectrophotometry and HPLC-UV chromatography techniques were employed. Average values and standard deviations of each reaction were calculated from three independent replicates; blanks and negative controls were prepared and measured at the same time. The catalytic constants of the enzyme were determined varying the concentration of EDCs until catalytic saturation; the transformation rate values were fitted to the Michaelis–Menten equation. In this work, with the only purpose of comparison among the catalytic profile of our cocktail and other reported laccases, a kinetic parameter *K*_app_ was defined as apparent for *K*_m._

#### UV–Vis Qualitative Analysis

The enzymatic treatment was carried out at room temperature in 5 mL reaction mixture containing 10 mg L^−1^ of each analyte, 10 % (*v*/*v*) buffer McIlvain pH 5 and 100 U L^−1^ (50.3 U mg ^−1^) of laccase; then the solution was vortex-mixed briefly for homogenizing and sheltering from light. To detect changes in the absorbance spectrum in a range of 200–500 nm, aliquots of 3 mL were taken from each treatment and blank of the analyte (without laccase treatments). The aliquots were measured in quartz cells at time 0, 2, 6 and 48 h using a Hach DR 500 spectrophotometer. After monitoring the maximum absorption wavelength, the parameters for the qualitative analysis by HPLC-UV were established.

#### Quantitative Analysis by HPLC-UV

Determination of EDCs was obtained through a HPLC system coupled to an UV–Vis detector (Agilent Technologies) and a reverse-phase column Agilent Eclipse XDE-C18 150 × 4.6 mm, 5 μ. A reverse-phase column Agilent Eclipse XDE-C18 150 × 4.6 mm, 5 μ, was used for the chromatographic measurements. The final reaction mixture was performed at 25 °C in 1 mL vial containing 10 mg L^−1^ of each analyte, 10 % (*v*/*v*) buffer McIlvain pH 5 and 100 U L^−1^ (50.3 U mg^−1^) of laccase; then the solution was vortex-mixed briefly for homogenizing and sheltering from light. The enzymatic treatments were measured by triplicate. An injection volume of 20 μL and 1 mL min^−1^ gradient elution by means of (A) acetonitrile (ACN) and (B) 10 mM phosphate buffer (pH 3.5) were applied. The gradient program was set as follows: 0–11 min, 25 % (A); 11–23 min, 95 % (A) and 23–30 min, 25 % (A). BPA, NP, TCS and EE2 were detected at three wavelengths, 206, 290 and 275 nm. The chromatographic analysis was carried out up to 12 h and the extent of the reaction was estimated by the decrease of the corresponding analyte peak analyzed by HPLC-UV chromatography technique and quantified using a calibration curve.

## Results and Discussion

### Enzyme Characterization

According to Ramírez-Cavazos et al. (2014b), the laccase isoforms produced by *P. sanguineus* in tomato juice medium and present in the crude extract show a relative activity of 65 % at 25 °C, increasing up to 100 % at 40 °C; the optimal pH values were observed in the acidic region. Table 1 summarized the pH range where laccase exhibit relative activity ˃85 % with 2,2′-azino-bis (3-ethylbenzthiazoline-6-sulfonate (ABTS); 2,6-dimethoxyphenol (DMP) and guaiacol (+) as substrates at 25 °C. Based on these results, a temperature of 25 °C and pH 5 were set to perform the degradation profiles of micropollutants since laccases still maintain very high catalytic activity; at the same time, it avoids the use of severe sample pretreatments (e.g. thermal or acidic process) which involves changes in matrix nature (water/groundwater samples).

**Table 1:** pH range where laccase isoforms exhibit a high relative activity and stability by using different substrates

pH range (relative activity ˃85 %)			
	ABTS	DMP	Guaiacol
Lac I	2–4	3–5	3.5–5.5
Lac II	2–3	2–4.5	3–5

### Qualitative Analysis by UV–vis Absorption Spectrophotometry

**Fig. 2:**
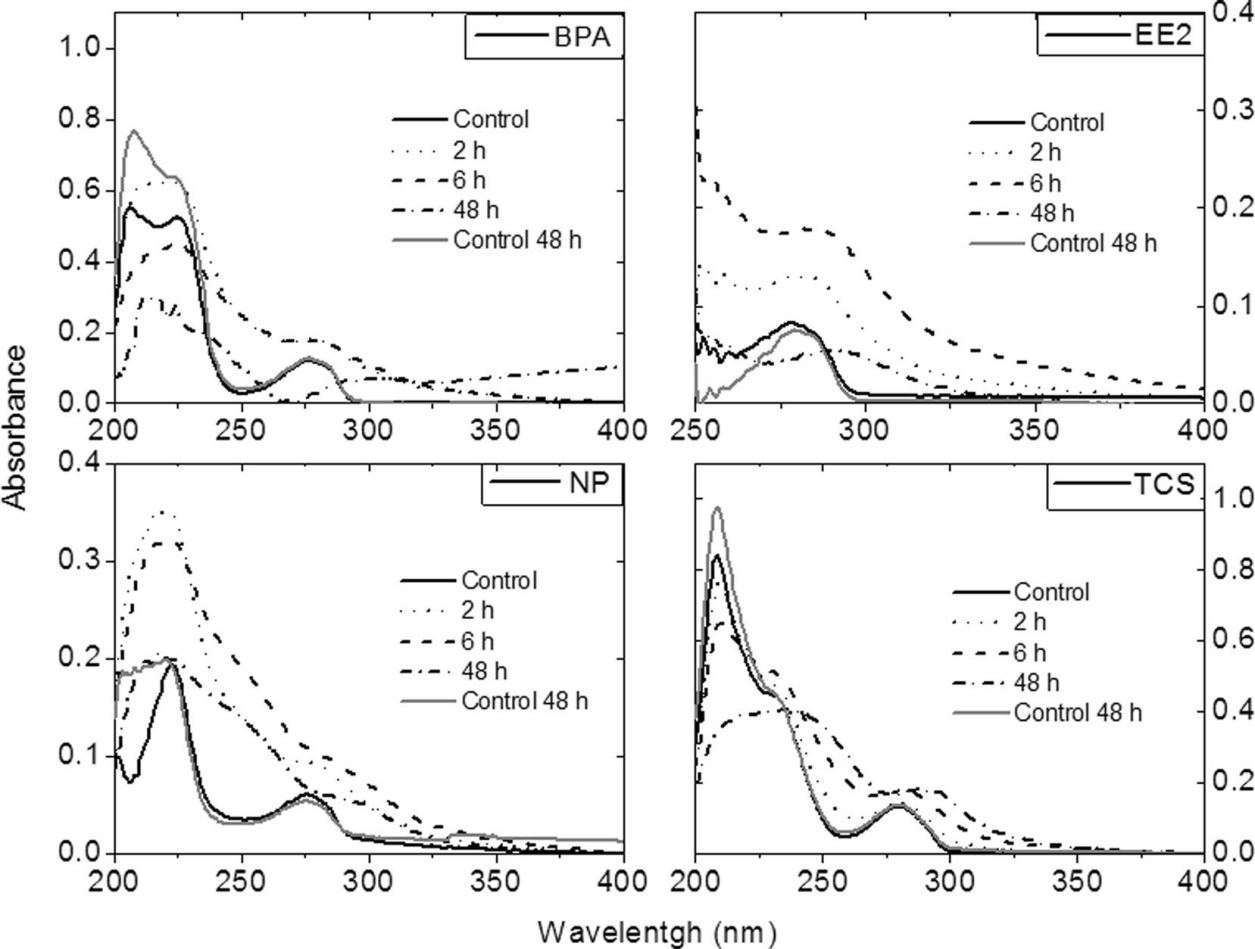
UV-spectroscopic analysis for enzymatic treatment by using 100 U L^−1^ laccase cocktail at pH 5 and 25 °C

Figure 2 shows the spectroscopic analysis by UV absorption for the selected EDCs before and after enzymatic treatment for their biotransformation. As the figure shows, changes in UV-spectra were apparent for all analytes after 2 h of treatment with laccase cocktail. It is noteworthy that spectra of blank analytes (controls) showed no significant changes after 48 h of monitoring. Bathochromic shifts were observed by the displacement of absorption bands around 275 nm to longer wavelengths (red shifts), and hypochromic shifts were recorded by the reduction in the absorbance intensity around 275 nm for EE2 and total disappearance for BPA. Bands around 275–290 nm are characteristic for phenolic compounds, indicating a clear biotransformation of these EDCs by laccase cocktail. Absorption bands were also observed at 300, 250 and 229 nm for BPA, NP and TCS, respectively, but not for EE2. For this EDC, a spectral saturation was observed nearly to 200 nm (not shown in Fig. 2). An explanation for all these new bands can be the possible formation of by-products, which possess a different spectral behavior. The preliminary UV-absorption study shows the potential role of this cocktail in the biotransformation of EDCs.

### Biodegradation Study of *EDCs* by Laccase Cocktail

**Fig. 3:**
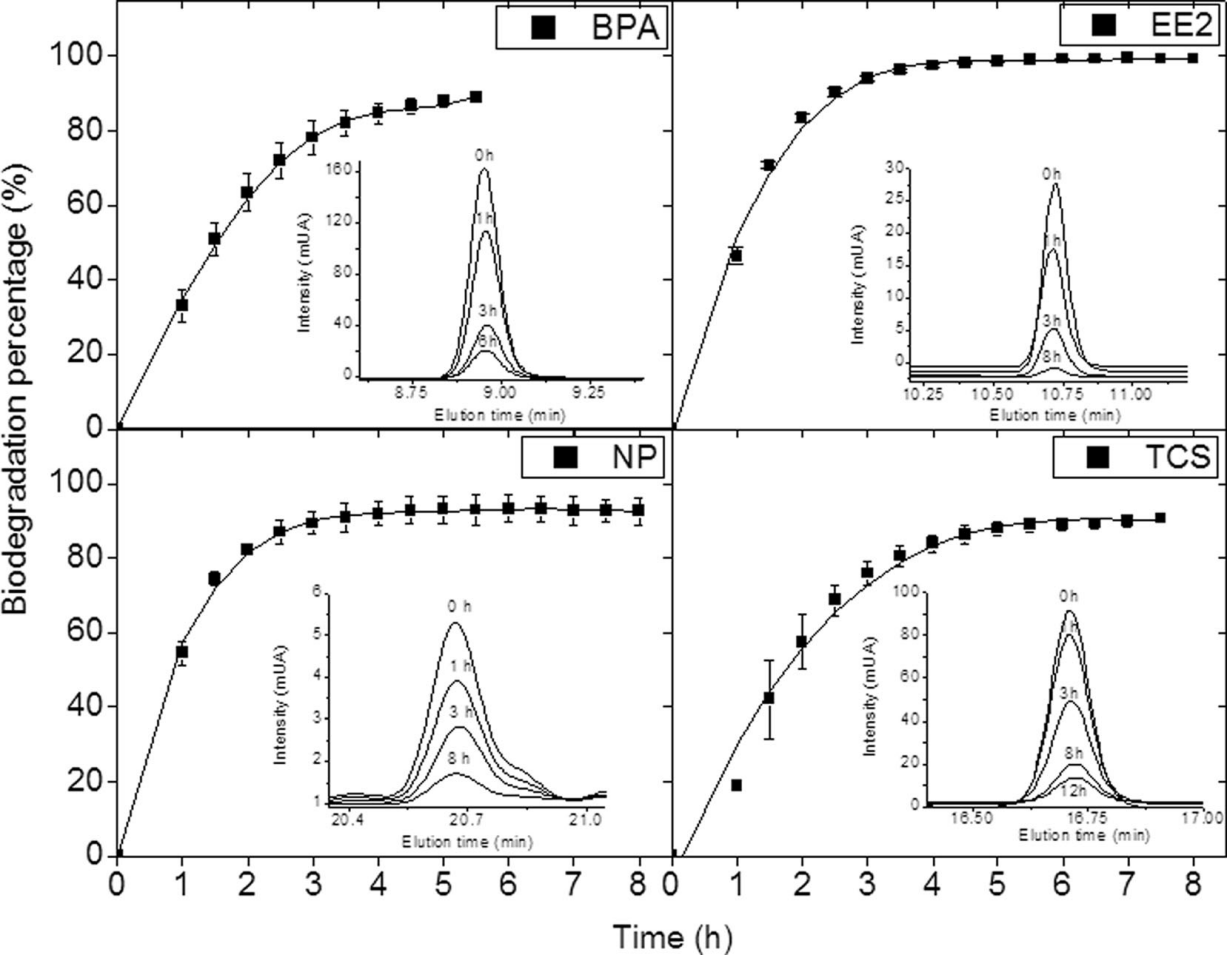
Biodegradation profile of 10 mgL^−1^ synthetic samples for BPA, EE2, NP and TCS by using 100 U L^−1^ laccase cocktail at pH 5 and 25 °C. Each graph presents the maximum areas of peak taken from the respective chromatograms at different times of enzymatic treatment

A quantitative analysis was performed in order to determine the biotransformation percentage of target EDCs by the laccase cocktail. Figure 3 shows the biodegradation profiles for each analyte due to enzymatic treatment as well as the chromatograms corresponding to the peak of interest at different times of enzymatic treatment.

A decrease in the signal intensity is observed in chromatograms, which indicates the biotransformation of the EDCs. It is noticed that around 5.0–5.5 h of enzymatic oxidation are enough to achieve a biodegradation higher than 89 % for all the analytes. These results are consistent since UV-absorption spectra of EDCs show significant changes after 6 h of treatment (shown in Fig. 2). The slight differences among EDCs in biotransformation percentage can be attributed to their chemical structures (see Fig. 1). Since laccase is an oxidoreductase which couples the one electron oxidation of phenolic substrates, the presence of electron donating functional groups (EDFG) or electron withdrawing functional groups (EWFG) play an important role in reactivity (Yang et al. 2013; Nguyen et al. 2013). According to Yang et al. (2013), EDFG such as hydroxyl (−OH), amines (−NH_2_), alkoxy (−OR), alkyl (−R) and acyl (−COR) groups induce the electrophilic attack by oxygenase enzymes which generates oxidation of molecules (Tadkaew et al. 2011). Moreover, the presence of EWFG reduces the efficiency of enzymes for attacking the analytes since groups such as amide (−CONR_2_), halogen (−X) and nitro (−NO_2_) produce an electron deficiency. The high biotransformation percentage of EE2 (100 ± 0.56 % in 5 h) can be explained by the presence of the hydroxyl group in the aromatic structure, thus making EE2 more susceptible to oxidation by laccase. In the case of NP, the percentage was 93 ± 2.93 % after 6 h of enzymatic treatment. This compound also features hydroxyl and alkyl groups in the aromatic structure which can contribute to its degradation. The next compound with higher biodegradation percentage was TCS (90 ± 0.94 % in 5.5 h). A particular characteristic of TCS is the presence of both EDFG and EWFG, in spite of the effect of chlorinated groups (EWFG), it seems that the strong electro-donor hydroxyl group makes this molecule appropriate for the laccase oxidation. BPA is another polyphenolic molecule with EDFG, containing two –OH groups and two −R groups; after enzymatic treatment, it presented a 89 ± 1.05 % of biotransformation. As reported previously by Cabana et al. (2007a), TCS were eliminated to a lesser degree, compared with NP. In this work, TCS and BPA were less susceptible to degradation by laccase cocktail than NP and EE2. Studies of enzymatic treatment with commercially available laccase from *Trametes versicolor* report fast enzyme inactivation after reacting conditions with BPA. This is due to the interactions between the radicals from BPA and the enzyme (Cabana et al. 2007b). According to Cabana et al. (2007b), the removal efficiency is directly related to the fungal strain, the culture conditions used and the nature of the xenobiotic molecules.

### Kinetic Results

**Fig. 4:**
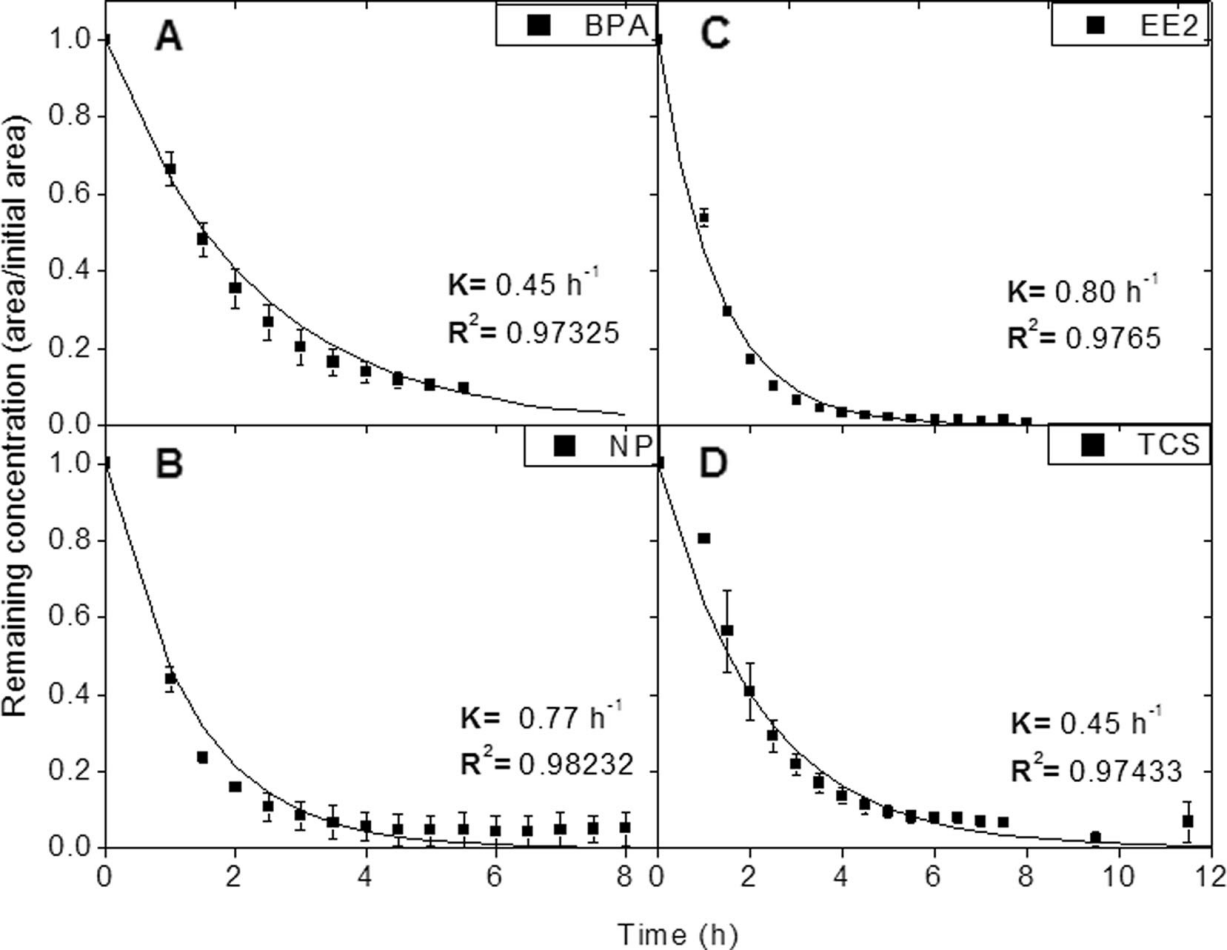
Biotransformation of EDCs catalyzed by laccase cocktail. Curves were fitted to first order reactions and kinetic rates (*k*) were calculated for the target analytes

Biodegradation rates were fitted to a first order reaction, according with the following equation:

1 $$ In\left[A\right]= In{\left[A\right]}_0-{k}^{\prime }t $$

where [*A*_0_] corresponds to the initial concentration of the analytes and [*A*] is the concentration at a determined time of the reaction; *k*′ is the adjusted rate constant used in the general model and *t* corresponds to time (in hours). For all the adjustments, an *R*^2^ > 0.97 was obtained. As can be seen in Fig. 4, *k*′ constants corresponding to the EDCs were obtained with a similar order of magnitude, in which BPA and TCS presented similar values (*k*′ = 0.45 h^−1^). According to these results, the reaction order is EE2 > NP > TCS = BPA which corresponds with the order of biotransformation percentages for the target analytes.

Some attempts to incorporate this novel technology include the construction of bioreactors (Lloret et al. 2013a, b), immobilization of laccases (Cabana et al. 2009; Torres-Duarte et al. 2012) and the assembly of enzyme electrodes for the biodegradation or monitoring of EDCs (Oguchi 2011). The vast majority of other studies have focused on the effects, quantification and bioremediation of just one EDC at a time. However, complex mixtures of EDCs are suspected of acting together on the endocrine system of organisms (Filby et al. 2007; LaFleur and Schug 2011), which requires a more extensive analysis. Another limitation is the high concentration of required enzyme for removing EDCs, which increases the cost of these processes.

Table *2* summarizes the studies that have been developed using laccases as free enzyme for biotransformation of target EDCs. Some of those works (Cabana et al. 2007c) present a comparison in the catalytic efficiency between free and immobilized laccases used for removal of pollutants; others employed crude extracts, purified or recombinant enzymes (Fukuda et al. 2001, 2004; Saito et al. 2003; Cabana et al. 2007a) in biodegradation treatments for synthetic (Kim and Nicell 2006b) or treated waters (Auriol et al. 2008).

**Table 2:** Catalytic parameters corresponding to the maximal biodegradation of EDCs achieved by different strains of laccases as free enzyme

Laccase strain	Analyte (ppm)	T (°C)	pH	Laccase concentration (U L^−1^)	Catalytic parameters				Mediator/additive	Removal		Comments	Ref.
					*V*_max_/*k*_T_	*K*_m_	*k*_cat_	*K*_cat_/*K*_m_		%	Time		
					(μM min^−1^)/(Ms^−1^)	(μM)	(s^−1^)	(mM^−1^ s^−1^)			(h)		
*Trametes versicolor*	EE2 (0.1 × 10^−3^)	25	7	800	53.4/9.28 × 10^6^	3.8	–	–	–	90	1	Free laccase with synthetic samples and municipal wastewater after conventional treatment processes in WWTP (treated and filtrated waters)	Auriol et al. (2007)
	EE2 (0.1 × 10^−3^)	25	7	20,000	–	3.8	0.01	2.23		100	1	Free laccase with synthetic samples and municipal wastewater after conventional treatment processes in WWTP (treated and filtrated waters)	Auriol et al. (2008)
	TCS (5.8)	25	5	3,000	0.92	24	–	–	–	100	4	Free laccase with synthetic samples, oxidation in presence and absence of mediator	Kim and Nicell (2006a)
	TCS (5.8)	25	5	3,000	60	180	–	–	ABTS^a^ (0.01 mM)	100	0.5		
	NP (22) NP (220)	25	5	0.1 mg/mL	–	420	–	–	–	100 60	1.5	Free laccase with synthetic samples	Catapane et al. (2013)
	EE2 (3)	30	4.5	600	–	–	–	–	HBT^b^ (0.2 mM)	100	8	Free laccase reaction under stirring at 150 rpm	Suzuki et al. (2003)
	NP (50) NP (50) BPA (50) BPA (50)	30	4.5	100 100	– – – –	– – – –	– – – –	– – – –	– HBT^b^ (0.2 mM) – HBT^b^ (0.2 mM)	60 78 70 97	1 1 1 1	Free laccase reaction under stirring at 150 rpm	Tsutsumi et al. (2001)
	BPA (27.4)	25	5	300	42.7	690	–	–	–	92	2	Free laccase with synthetic samples	Kim and Nicell (2006c)
	BPA (27.0)	25	5	300	42.7	690	–	–	PEG^c^ (1.5 μM)	95	2		
	BPA (27.0)	45	5	150	–	–	–	–	–	68	1	Free laccase with synthetic samples	Kim and Nicell (2006b)
	BPA (27.0)	25	5	150	–	–	–	–	– ABTS^a^ (100 μM) HBT^b^ (100 μM) SA^d^ (100 μM) VLA^e^ (100 μM) TEMPO^f^ (100 μM)	58 97 56 60 72 52	1 1 1 1 1 1		
*Coriolopsis gallica UAMH 8260*	TCS (25)	25	4.5	0.5–8 U g^–1^	–	970	1.5	1.5	–	100	n.r.^g^	Free laccase with synthetic samples	Torres-Duarte et al. (2012)
	NP (25)	25	4.5	0.5–8 U g^−1^	–	420	17.5	42.2	–	100	n.r.^g^		
	BPA (5.0)	25	4.5	0.5–8 U g^−1^	–	670	13.9	20.7	–	85	18		
*Trametes villosa*	BPA (500)	60	6	1,500	–	14 100	0.98	–	–	100	1	Recombinant laccase produced in *Aspergillus oryzae*	Fukuda et al. (2001, 2004)
*Strain I*-*4 of the family Chaetomiaceae*	BPA (1141)	40 40	7 7	50,000	–	10,000	14	1.4	–	99	3	Free laccase with synthetic samples	Saito et al. (2003, 2004)
	NP (1102)			50,000	–	5,000	1	0.2	–	99	6		
*Coriolopsis polyzona*	BPA (5) NP (5) TCS (5)	40 50 50	5	10 1 100	–	–		–	–	100 100 65	4 8 8	Crude enzyme preparation	Cabana et al. (2007a)
*Pycnoporus sanguineus* *Sp. CS43* *Laccase cocktail* (*LacI*/*LacII*)	BPA (10.0)	25	5	100	14.98	481.9	–	–	–	89	5.5	Free laccase in synthetic and groundwaters samples	Present study
	EE2 (10.0)	25	5	100	2.17	32.0^h^	–	–	–	100	5		
	NP (10.0)	25	5	100	23.84	73.6^h^	–	–	–	93	5.5		
	TCS (10.0)	25	5	100	7.76	302.5^h^	–	–	–	90	5.5		

^a^ *ABTS* 2,2 0 -azino-bis-(3-ethylbenzthiazoline-6-sulfonic acid)

^b^ *HBT* 1-hydroxybenzotriazole

^*c*^ *PEG* polyethylene glycol

^*d*^ *SA* syringaldehyde

^*e*^ *VLA* violuric acid

^*f*^ *TEMPO* 2,2,6,6-tetramethoxypiperidine 1-oxyl

^g^ *n.r.* not reported

^h^Kapp

The overall effect of the biodegradation method proposed in this study showed comparable removal percentages with respect to previous works using other fungal laccases (see Table *2*). In the case of BPA transformation, the calculated *K*_app_ value shows that there is no strong affinity for this compound in comparison with the *K*_m_ value for other EDCs. The removal percentage achieved in this study (89 %) is comparable with the results reported by Torres-Duarte et al. (2012; 85 %) and is even better than previous treatments at 25 °C that reported the use of mediators like 1-hydroxybenzotriazole (56 %), syringaldehyde (60 %) and violuric acid (72 %; Kim and Nicell 2006b). It is important to notice that the use of mediators are very common in enzymatic treatments; however, it adds complexity to the system by augmenting the amount of by-products after the biotransformation of EDCs. Moreover, they are expensive and some has already demonstrated to be toxic, such as PEG (Kim and Nicell 2006c; Murugesan et al. 2010). The best results for BPA degradation (˃90 %) were obtained only under high temperatures of reaction (Fukuda et al. 2001, 2004; Cabana et al. 2007a) and using elevated laccase concentrations (Saito et al. 2003, 2004). For EE2, the biodegradation percentage obtained in this study was 100 % meanwhile Auriol et al. (2007) achieved a biodegradation of 90 % for (100 ng L^−1^) after 1 h of treatment with 800 U L^−1^ of laccases from *T. versicolor*, with a Km of 3.9 μM. Then, Auriol et al. (2008) obtained 100 % of removal under the same conditions but using a higher laccase concentration, 20,000 U L^−1^. Finally, Suzuki et al. (2003) also obtained an EE2 removal of 100 %, but the temperature of reaction was at 30 °C and 1-hydroxybenzotriazole was added as mediator. For NP biotransformation, it can be noted that the highest affinity for this substrate was obtained with laccase cocktail from *P. sanguineus* CS43, with a *K*_app_ value of 73.6 μM and obtaining 93 % of removal. A similar study to the present work (i.e. by using free laccase, besides culture supernatant) was developed with laccase obtained from the I-4 strain from the family *Chaetomiaceae* (Saito et al. 2004), in which after 6 h, 100 % of NP (1,102 ppm) was removed but using high amounts of laccase (50,000 U L^−1^). Lower results were obtained by Tsutsumi et al. (2001), even using HBT as mediator (at 30 °C). In the case of TCS, a removal of 90 % was achieved in this work, only surpassed by works that obtained complete removal employing high amounts of enzyme (Kim and Nicell 2006a) and acidic conditions (Torres-Duarte et al. 2012). Under similar conditions of treatment, this study demonstrated that the overall degradation effect of the enzymatic reaction seemed to be comparable and in some cases, better regarding previous works presented in Table *2*. As we reported in a previous study (Ramírez-Cavazos et al. 2014b), the laccase isoforms, present in the crude extract used in this study, are thermostable and highly active up to 70 °C; however, the aim of this work was to develop a sustainable and low-cost water treatment; thus, the reaction was maintained under mild conditions.

## Application on Groundwater Matrices

Due to the high redox potential of free laccases of a cocktail from *P. sanguineus* CS43 (data not shown), these enzymes can be implemented as an efficient method for the purification of water. In this study, the enzymatic activity was evaluated in real groundwater samples. The samples used in the study were obtained from La Paz Valley, Mexico.

### Experimental Site

La Paz Valley, a desert area located in northwestern Mexico, has a population of 283,000 habitants (Ojeda-Lavin 2012); the water supply for this region is obtained from groundwater resources (87 %). A recent study has revealed a high salinity in groundwaters (287–5352 mg L^−1^), showing elevated concentrations of chlorides (90–2,960 mg L^−1^) mainly from NaCl and CaCl; it has a pH range between 6.8 and 8.3. The presence of Na^+^, K^+^, Ca^2+^, Mg^2+^, Cl^−^, F^−^ HCO_3_^−^ and SO_4_^2−^ and SiO_2_ ions suggests water–rock interactions, ion exchange and seawater intrusion, while fluoride concentration is related to hydrothermal fluids; NO_3_^−^ and SO_4_^2−^ are related to anthropogenic components. Table 3 shows field parameters and chemical constituents of groundwater samples.

**Table 3:** Field parameters and major ion concentrations in groundwater of selected wells in La Paz

Sample no.	Water use	Land use	Temp	SEC	pH	Ca^2+^	Mg^2+^	Na^+^	K^+^	Cl^−^	HCO_3_^−^	SO_4_	F^−^	SiO_2_
			(°C)	(μS cm^−1^)		(mg L^−1^)	(mg L^−1^)	(mg L^−1^)	(mg L^−1^)	(mg L^−1^)	(mg L^−1^)	(mg L^−1^)	(mg L^−1^)	(mg L^−1^)
LP-01	Urban	Urban area	28.9	893	6.8	51.0	16.5	67.5	4.07	159	166	37.1	<0.03	19.6
LP-03	Urban	Urban area	29.2	1,537	6.9	103.0	33.7	99.9	5.5	385	185	34.3	<0.05	21.9
LP-07	Urban	Loose topsoil	31.1	2,109	7.1	155.0	60.3	80.9	4.94	521	190	77.1	<0.1	30.7
LP-09	Urban	Dessert land	30.5	865	7.4	52.7	20	81.7	2.71	118	280	47.5	0.08	27.4
LP-13	Urban	Dessert land	31.8	1,155	7.6	43.6	10.1	141	5.5	188	250	53.5	1.47	39
LP-18	Agriculture	Cropland	31.0	5,100	7.0	335.0	186	391	9.12	1,400	578	195	<0.3	37.9
LP-22	Agriculture	Cropland	30.7	7,520	7.0	356.0	131	1,080	10.2	2,260	498	490	<0.3	45
LP-28	Urban-agriculture	Cropland	29.0	6,880	7.0	421.0	186	763	8.94	2,030	634	441	<0.3	47.9
LP-31	Multiple	Cropland	27.1	4,770	7.1	189.0	110	693	3.49	1,140	1,290	228	<0.3	43.5
LP-32	Agriculture	Cropland	29.0	2,589	7.2	237.0	55.9	134	3.59	731	325	50.3	<0.1	33.2
LP-35	Urban	Urban area	31.3	2,751	7.2	255.0	74.5	102	2.97	793	276	64.3	<0.1	29.3
LP-38	Agriculture	Cropland	29.5	799	7.8	44.5	20.7	59.8	2.05	164	186	11.5	0.07	36.2
LP-39	Agriculture	Cropland	29.9	683	7.6	33.3	15.2	58.4	2.25	95.6	218	14	0.06	35.9
LP-40	Urban	Urban area	28.7	8,920	7.2	658.0	344	583	14	2,960	984	243	<0.5	36.2
LP-44	Urban	Urban area	30.2	630	7.2	33.5	15.6	50.1	1.54	89.7	198	14.5	0.2	25.8

### Biotransformation Behavior of EDCs in Groundwater Samples

**Fig. 5:**
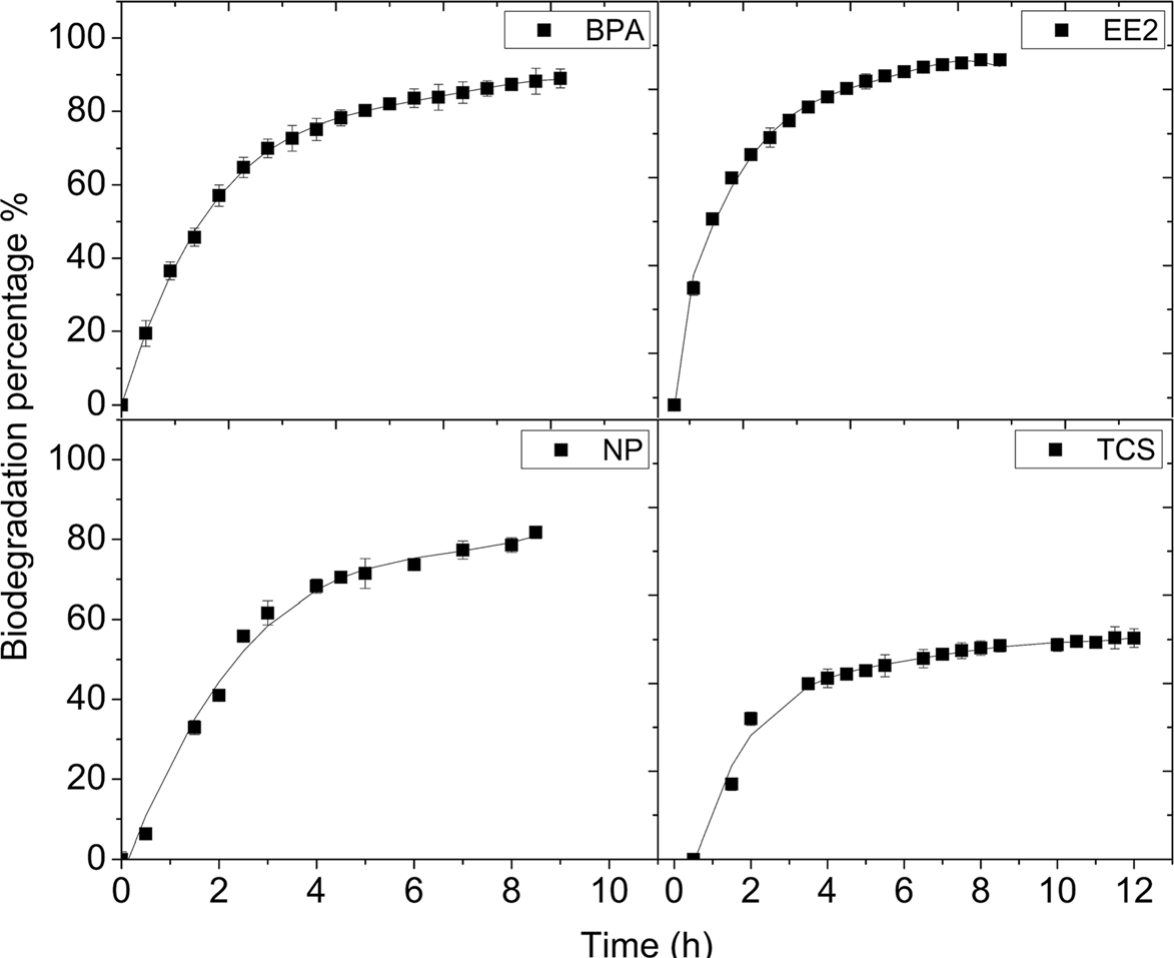
Biodegradation profiles of BPA, EE2, NP and TCS by using a representative bulk sample from groundwater. A concentration of 100 U L^−1^ laccase cocktail from *P. sanguineus* CS43 and 10 mg L^−1^ EDCs at pH 5 and 25 °C

Since the majority of studies regarding the biodegradation of EDCs have been carried out by using synthetic (Kim and Nicell 2006b) or treated waters (Auriol et al. 2008), there are scarcely studies that monitor the enzymatic activity in a real matrix with the presence of denaturant substances of laccases (e.g. organic solvents, heavy metals, ions, etc.). In this context, the purpose of this work was also to evaluate the laccase efficiency in a complex matrix for the biotransformation of the BPA, NP, EE2 and TCS. To construct a representative bulk sample, a mixture was assembled with all samples presented in Table 3 by adding equal aliquotes of each one. The reaction mixture was prepared by spiking the bulk groundwater sample with appropriate amounts of each analyte (final concentration of 10 mg L^−1^) and treated under the conditions described in Section 2.4. Results of biotransformations are displayed in Fig. 5.

As Fig. 5 shows, there is a significant decrease in the biotransformation percentage of TCS (55 ± 2.33 %), which in fact was the analyte with the lowest rate of biodegradation in synthetic samples. For the rest of the analytes, the matrix did not have strong influence in the laccase catalytic ability; BPA 87 ± 1.48 %, EE2 94 ± 0.63 % and NP 81 ± 1.53 %. Although in general a reduction in the removal percentage is observed for all EDCs in comparison with synthetic samples, it is noteworthy that the target compounds are not affected in the same way. This performance can be explained in terms of the variety of components (see Table 3) which are normally found in wastewater effluents and groundwaters by soil filtration. The presence of many ions from soil and antropogenic sources interact with laccases and cause interferences on the biodegradation of analytes. Chloride (Cl^−^), halide anions (F^−^ and Br^−^) and hydroxide anion (OH^−^) have been reported to bind to the T2 Cu of laccase and interrupt the internal electron transfer between T1 and T2/T3 or to bind near the T1 active site, blocking the access of the substrate to T1 Cu (Margot et al. 2013). Our previous study (Ramírez-Cavazos et al. 2014b) related the presence of some of these components with the decrease in the activity of laccase isoforms in the crude extract used in this work (see Table *4*). The work of Kim and Nicell (2006b) also proved that the presence of these components is related with the decrement of conversion by laccase from *T. versicolor*. Other interferences, such as cyanide (originated by the plastic industry), cause the dissociation at the copper ion from the enzymatic active site, as well as calcium, cobalt and zinc chlorides, which tend to interfere by hydrogen bonding (chaotropic effect; Cabana et al. 2007b).

**Table 4:** Effect of inhibitors on purified laccase activities

	IC_50_ (mM)		Complete inhibition (mM)	
Substrates	Lac I	Lac II	SD Lac I	SD Lac II
NaF	0.08	0.02	16	8
NaCl	65	14	˃2,000^a^	˃2,000^a^
NaN_3_	6.20E-06	6.90E-07	16	16
Na_2_SO_4_	˃800^b^	˃800^b^	˃800^a^	˃800^a^

Laccase activity was measured using ABTS as the substrate at pH 3 (modified from Ramírez-Cavazos et al. 2014b)

^a^Values refer to the respective highest concentration tested where a complete inhibition was not observed

^b^Values refer to the respective highest concentration tested where 50 % inhibition was not observed

By taking advantage of the thermostability of *P. sanguines* CS43 laccase (Ramírez-Cavazos et al. 2014b), the degradation rates obtained in groundwater samples would significantly improve by increasing the reaction temperature, above 30 °C in the biodegradation treatment. The implementation of immobilization methods in different materials can be employed to enhance the biodegradation results, as well.

## Conclusions

Biotransformation of BPA, EE2, NP and TCS using a laccase cocktail from *P. sanguineus* CS43 was studied and compared in both synthetic and real groundwater samples. A removal higher than 89 % was achieved for all selected analytes in synthetic samples, which were achieved using free laccase enzyme and avoiding the use of mediators. In terms of the biotransformation on real groundwater samples, a decrease in degradation percentages caused by the interaction of the ions present in the complex matrix were clearly observed. This study reveals the high biocatalytic efficiency of this cocktail composed by LacI and LacII for the removal of common EDCs with low amounts of laccase activity (100 U L^−1^) and treatment time in comparison with other studies. Consequently, laccase cocktail from *P. sanguineus* strain CS43 represents a promising alternative for biotransformation systems with operational advantages such as less purification steps. To enhance the biotransformation process, further work will be focused on testing this enzyme under conditions above 25 °C. Immobilization methods as well as scaling-up bioreactors using these biomaterials can represent an opportunity to study and to design novel biodegradation technologies.
